# The Risk of Hemorrhage and Shunt Occlusion After Tonsillectomy in a Glenn Circulation Patient With Blalock-Taussig Shunt Dependence: A Case Report

**DOI:** 10.7759/cureus.78682

**Published:** 2025-02-07

**Authors:** Tokimitsu Hibino, Yusuke Okui, Yoshie Toba

**Affiliations:** 1 Department of Anaesthesiology, Seirei Hamamatsu General Hospital, Hamamatsu, JPN

**Keywords:** blalock-taussig shunt, glenn procedure, perioperative anticoagulation, shunt occlusion, tonsillectomy

## Abstract

Tonsillectomy is among the most common procedures performed on children. Children with congenital heart disease sometimes undergo tonsillectomy to reduce pulmonary arterial pressure. These children can have low cardiac function and low oxygen saturation (SpO_2_) and pose particular challenges to the anesthesia team. In this article, we report our experience of anesthetic management of a child with post-Glenn procedure and Blalock-Taussig shunt dependence who underwent a tonsillectomy. To avoid postoperative hemorrhage, anti-coagulants/anti-platelets were discontinued five days before surgery. After discontinuation of these medications, we administered heparin until five hours before surgery. Anti-coagulants/anti-platelet medications were resumed on postoperative day 15 when epithelialization of the surgical wound was achieved. This strict management helped avoid hemorrhage but may have induced the subsequent postoperative Blalock-Taussig shunt embolic occlusion, which required reconstruction. In our case, we gave priority to the prevention of postoperative hemorrhage and delayed the restart of anticoagulation therapy, although we now believe that the risk of thrombosis should be given more attention and anticoagulation treatment should be restarted as soon as possible.

## Introduction

Tonsillectomy is one of the most frequently performed procedures on children, with over 500,000 tonsillectomies performed annually in the United States [1]. The most common indications for tonsillectomy include infections and upper airway obstructions [2], but patients with pulmonary hypertension may also benefit from the procedure, as it has been shown to reduce pulmonary arterial pressure (Yilmaz et al.) [3]. Patients with congenital heart disease (CHD) are vulnerable to pulmonary hypertension and, therefore, sometimes undergo tonsillectomy [4].

Idiopathic pulmonary arterial hypertension and heritable pulmonary arterial hypertension are the most common causes of pulmonary hypertension in children [5]. Together, they account for 56% of the total. CHD-related pulmonary hypertension accounts for 36%, making it the second most common cause of pulmonary hypertension [5].

In CHD, pulmonary hypertension is usually secondary to left-right shunts or posterior capillary hypertension due to left heart obstructive disease [6]. Of these, complex lesions, such as atrioventricular septal defects and arterial trunk disease, often cause pulmonary hypertension at a young age [7]. And those complex lesions include cyanotic heart disease (CCHD).

In CCHD, venous and arterial blood mix in the heart due to a defective hole, obstructive lesion, or shunt in the heart and/or great arteries. In healthy individuals, venous blood is originally pumped from the right ventricle to the lungs, and arterial blood, oxygenated in the lungs, is pumped from the left ventricle to the body. The flow of blood at this point is a simple one-way flow. In CCHD, on the other hand, venous and arterial blood are mixed and pumped from the heart to the lungs or the body. In other words, part of the venous blood returned from the body goes to the body again; part of the arterial blood returned from the lungs goes to the lungs again. Since blood from the heart is pumped to both the body and the lungs, this is a parallel condition.

In parallel conditions of CCHD, pulmonary and systemic perfusion are determined by the balance between pulmonary and systemic vascular resistance. If pulmonary vascular resistance increases, pulmonary blood flow decreases and systemic blood flow increases. When systemic vascular resistance increases, systemic blood flow decreases and pulmonary blood flow increases. Therefore, balancing pulmonary and body vascular resistance is crucial in the management of parallel conditions of CCHD [8]. Complicated pulmonary hypertension can disrupt this balance between pulmonary and systemic vascular resistance, making the management of CCHD difficult. To improve cyanosis within the single ventricle, a bidirectional Glenn surgical technique is used to create superior cavopulmonary anastomosis as a precursor to further treatment such as Fontan. In some cases, these patients may also require a tonsillectomy to improve pulmonary hypertension [4].

Postoperative hemorrhage is one of the most common adverse events of tonsillectomy, affecting approximately 0.5% to 18% of patients [9]. Life-threatening postoperative hemorrhage in pediatric patients can be as high as 5% [10]. It is bimodal, with primary hemorrhage occurring within 6 hours after surgery and secondary hemorrhage occurring 7 to 10 days after surgery [11]. Pediatric CHD patients who take anti-platelet and anti-coagulant agents may be at a higher risk of postoperative hemorrhage.

Administering general anesthesia to pediatric patients with cyanotic CHD (CCHD) who suffer from hemorrhage poses many challenges to the anesthetic team. If the hemorrhage requires surgical intervention, general anesthesia (GA) is almost always necessary. Young infants with CCHD have relatively low SpO_2_ and cannot tolerate even slight hypoxemia so preparation for the risk of arterial oxygen desaturation during lengthy intubation becomes crucial. Preoxygenation in these situations is critical. Rapid administration of oxygen through masks or intubation, however, may be inhibited by spit or vomited blood, which is often experienced by these patients. Preventing this situation is paramount to a good outcome.

In this article, we present the anesthetic management of a tonsillectomy for a post-Glenn procedure infant with underlying pulmonary hypertension. To our knowledge, there is no literature on the duration of discontinuation of anti-coagulation/antiplatelet therapy in the perioperative period in patients with Blalock-Taussig shunt (BTS). Therefore, our priority was to reduce the risk of postoperative bleeding. We resumed anticoagulant/anti-platelet therapy on postoperative day 15 because the wound was considered to be completely epithelialized and the risk of postoperative bleeding was considered to be eliminated. There was, however, graft occlusion at the BTS, which required reconstruction. Retrospective observation did not reveal when the obstruction occurred. We discuss perioperative options for anti-coagulant/antiplatelet therapy in the present case.

## Case presentation

A 2-year-11-month-old boy with an atrioventricular septal defect and underlying pulmonary hypertension presented for single ventricular repair. He was born by cesarean section and though his oxygen saturation (SpO_2_) sometimes fell below 95%, he was discharged with no further examination. He exhibited poor weight gain and cardiac murmur and was diagnosed with an atrioventricular septal defect underlying pulmonary hypertension by trans-thoracic echocardiogram at four months of age. Given his unbalanced ventricles and abnormal morphology of the atrioventricular valve, biventricular repair was replaced with single ventricular repair. At five months of age, he underwent pulmonary artery banding to control excessive pulmonary arterial flow. After pulmonary artery banding, his SpO_2_ and blood pressure suddenly decreased while he was in the intensive care unit (ICU); an event that was most likely a result of a pulmonary hypertensive crisis.

**Figure 1:**
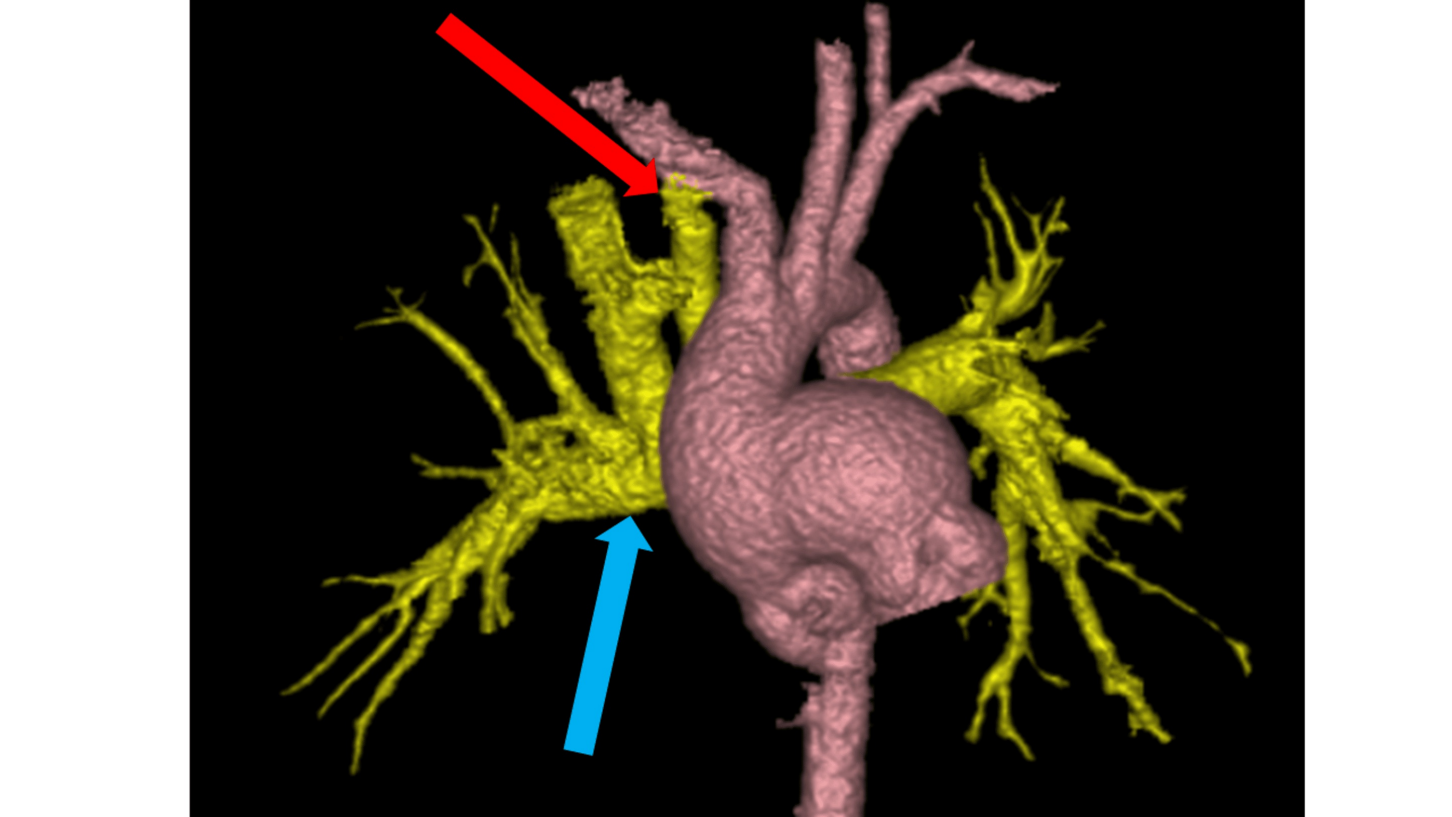
Modified Blalock-Taussig shunt after the Glenn surgery The red arrow indicates the BTS graft. The blue arrow indicates the Glenn anastomosis. Due to high pulmonary vascular resistance, the Glenn circulation could not supply sufficient pulmonary blood flow. The Glenn anastomosis alone could no longer maintain oxygenation. Therefore, a BTS was urgently constructed. BTS improved pulmonary blood flow. Postoperative angiography showed that blood flow from the superior vena cava was primarily to the right pulmonary artery while blood flow from the BTS was to the left pulmonary artery. BTS: Blalock-Taussig shunt

His pulmonary hypertension was treated with tadalafil, a phosphodiesterase 5 inhibitor, and ambrisentan. Inhibition of phosphodiesterase 5 inhibits the breakdown of cyclic GMP and promotes vascular smooth muscle relaxation in pulmonary arteries [7]. Ambrisentan is a competitive inhibitor of the endothelin-1 receptor that inhibits the vasoconstrictor and cell growth effects of endothelin-1 [7]. With these oral treatments, pulmonary artery pressure decreased to 14 to 16 mmHg and pulmonary vascular resistance (PVR) to 2.0 Wood Unit. Improvement in his pulmonary hypertension allowed him to gain enough weight and he underwent the Damus-Kaye-Stansel and Glenn procedure at 2-years-3-months of age. After the procedures, his PVR increased dangerously which resulted in failing circulation. Although he was intubated and on artificial respiration with pure oxygen, his SpO_2_ was only 60%, which may have been due to low pulmonary artery flow from the high PVR. To increase pulmonary artery flow, he underwent emergency BTS construction (Figure 1), but his circulation was still unstable and he was transferred to ICU under extracorporeal membrane oxygenation (ECMO). He gradually recovered and was taken off ECMO on postoperative day three. He was extubated on postoperative day five and discharged with 2 L/min oxygen therapy through the nasal cannula on postoperative day 30. At that time, his SpO_2_ was 80%.

**Figure 2:**
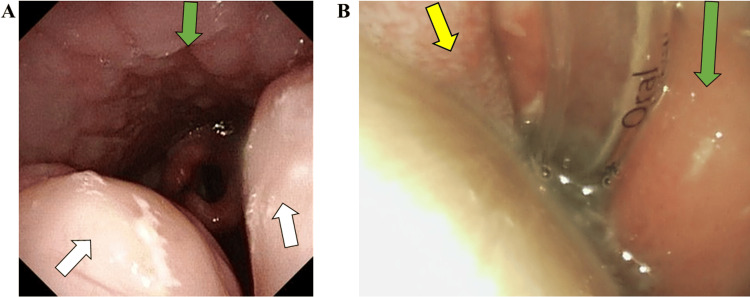
Pharyngolaryngoscope findings The green arrows indicate the pharyngeal wall. The white arrows indicate the palatine tonsil. The yellow arrow indicates the tongue. 2A: Bilateral tonsillar hypertrophy is evident. 2B: Day 2 after surgery. Pharyngeal edema was evident. Due to edema, the larynx and vocal cords were not visible, and plans for extubation were postponed. His Glenn circulation required high pressures due to high pulmonary vascular resistance. The superior vena cava pressure was high, averaging 17 mmHg, and the veins of the head and neck were constantly enraged. As a result, a slight increase in fluid volume caused facial swelling. We had expected edema at the surgical site after surgery, but the degree of edema was far beyond our expectations.

A few months later, at 2-years-11-months of age, he caught an upper respiratory infection that induced respiratory failure. He presented to the hospital with a cough, sputum, runny nose, and fever of 38 °C. Retractive breathing was observed, and the FilmArray® Respiratory Panel (BioFire Diagnostics, LLC., Utah, USA) was used to check for pathogenic viruses, but no pathogenic viruses were identified. Chest radiographs showed no evidence of pneumonia and enhanced pulmonary vascular shadows; findings suggestive of worsening heart failure. Blood cultures were negative, and sputum culture detected Moraxella catarrhalis. Based on symptoms and imaging findings, viral upper respiratory tract infection was the main diagnosis. Ampicillin sodium/sulbactam sodium 150 mg/kg/day was started for Moraxella catarrhalis and carbocisteine for phlegm. Furosemide 1 mg/kg/day was administered for suspected worsening heart failure. His SpO_2_ was as low as 65% and noninvasive respiratory support (high-flow nasal cannula (HFNC); Optifloe^TM^ Fisher & Paykel Healthcare Corp., Auckland, New Zealand) was started with 50% FiO_2_ and 20 L/min total flow. Despite the respiratory support, his SpO_2_ only improved to 70%. This desaturation could be attributed to upper airway obstruction and pulmonary hypertension, which in turn were implicated by tonsillar hypertrophy (Figure 2A). To address this problem, he was scheduled for tonsillectomy. As his tonsillectomy was considered high risk, a conference of pediatric cardiologists, cardiac surgeons, otolaryngologists, and anesthesiologists was held with a focus on patient safety. 

The cardiologist presented the patient’s cardiac history. The patient was post-Glenn procedure, in which the superior vena cava had been anastomosed to the pulmonary artery. The patient had pulmonary hypertension, which required high superior vena cava pressure for the Glenn circulation to flow. A cardiac catheterization performed 6 months earlier showed a high superior vena cava pressure of 17 mmHg compared to an inferior vena cava pressure of 10 mmHg. The cardiologist noted that venous return in the upper body may be impeded because of high superior vena cava pressure, so even a slight intravenous infusion would cause his face to swell, which would make intubation challenging. The cardiologist added that the risk of postoperative bleeding was considered higher than usual because of the stasis of the veins in the head and neck region. The cardiac surgeon, on the other hand, pointed out that the risk of BTS occlusion was high. Otolaryngologists stated that the procedure itself was simple and could be completed in a short time. However, postoperative bleeding was a possible complication and the risk could last approximately two weeks until the surgical wound was completely epithelialized. The anesthesiologists added that if postoperative bleeding occurred, induction of general anesthesia to deal with it could be fatal. Options for perioperative anticoagulation were discussed, but all of them recommended stopping warfarin and aspirin five days prior to surgery. The pros and cons of perioperative heparin bridging, as well as its timing, were discussed. Cardiac surgeons were reluctant to stop heparin because of the risk of BTS occlusion, whereas others were more concerned with postoperative bleeding and pushed for its discontinuation. Various opinions were expressed regarding the timing of resuming warfarin and aspirin administration such as from the first postoperative day, after extubation, or after epithelialization of the surgical wound was achieved.

After much discussion, the postoperative management plan was to administer a sedative with intubation for three days, along with fasting for a week for wound rest. To further reduce the risk of hemorrhage, aspirin, and warfarin would be replaced with heparin (250 Units/kg/d) from five days before the tonsillectomy until five hours before the surgery. We decided on a plan of not administering heparin postoperatively, but resuming warfarin and aspirin after epithelialization of the surgical wound was achieved.

General anesthesia was induced with midazolam, fentanyl, rocuronium, and atropine, maintained with remimazolam, fentanyl, and rocuronium. After the induction and intubation, dopamine was administered through a 5F central venous catheter (CVC) (ARROW® Pediatric Two-Lumen Teleflex Inc., Wayne, PA, USA) to stabilize his circulation. He underwent tonsillectomy without any adverse events. The operation took 32 minutes while anesthesia was 117 minutes. The infusion volume, amount of bleeding, urine volume, and total fluid balance were 298 mL, 3 mL, 313 mL, and －18 mL, respectively. He was transferred to the ICU under dexmedetomidine 0.6 μg/kg/min, without extubation (at our facility, dexmedetomidine is used primarily for sedation of children in the ICU). At the time, his SpO_2_ was 86% under pure oxygen.

**Figure 3:**
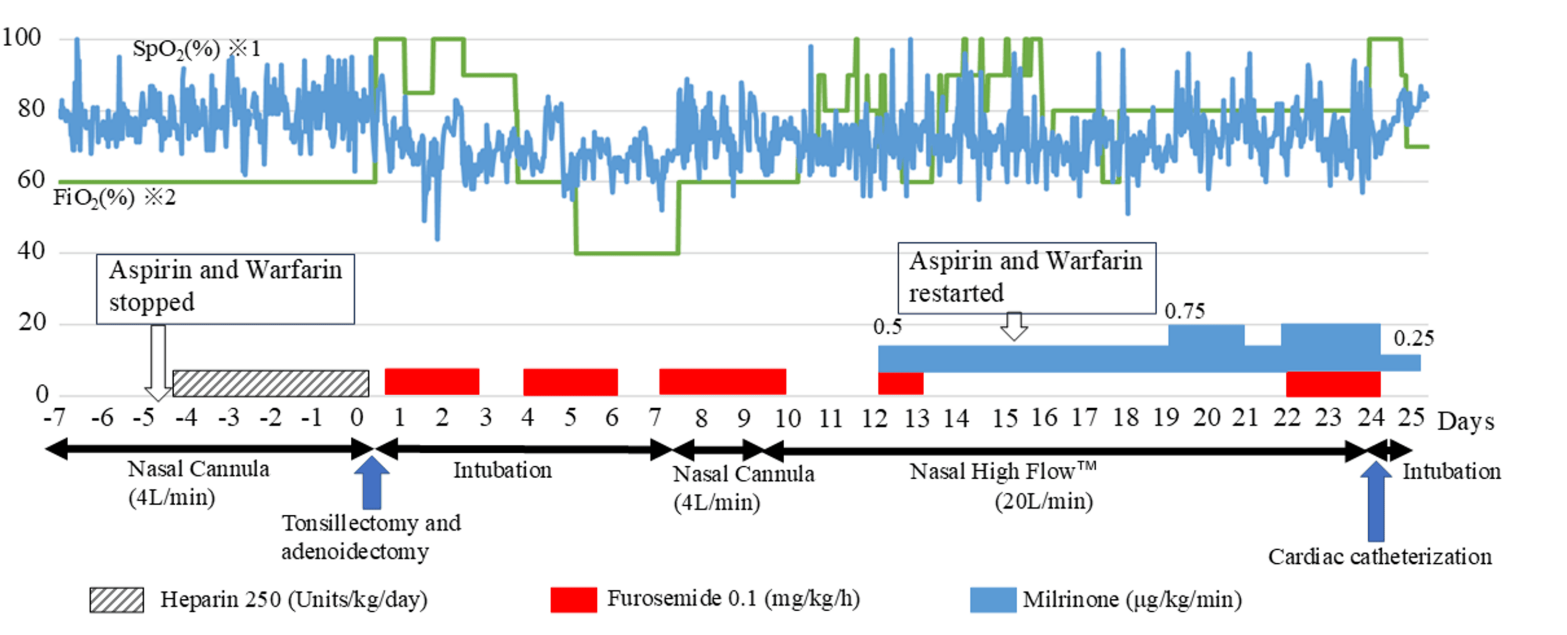
Perioperative SpO2 trends and modalities of respiratory assistance The graph shows the patient's perioperative FiO_2_ and SpO_2_. The vertical axis shows percentages; the horizontal axis shows the number of days, with the day of tonsillectomy as day 0. The boxes with white arrows indicate when to stop and restart aspirin and warfarin. The shaded horizontal bar indicates the duration of heparin administration. Red horizontal bars indicate the duration of furosemide administration. The blue horizontal bars indicate the duration of milrinone administration. The thickness of the blue bars indicates the dosing rate. The black two-way arrows indicate the modalities of respiratory support. Compared to the preoperative period, the FiO_2_ required to maintain the same level of SpO_2_ increased postoperatively. On postoperative days 1 to 9, furosemide was used aggressively to improve wound edema. After extubation, SpO_2_ remained lower than preoperatively and did not improve after 5 days of follow-up. To decrease pulmonary vascular resistance, milrinone was started on postoperative day 12. Preoperatively, the patient was oxygenated with a nasal cannula, FiO_2_ was 60% and SpO_2_ was 78.6 (±6.2) %. On postoperative days 8 and 9 after extubation, SpO_2_ was significantly lower 72.9% (±6.7) (p=0.0000149) with nasal cannula oxygenation and FiO_2_ of 60% the same as before surgery. On postoperative days 10 to 23, SpO_2_ was 72.3 (±6.9) %, lower than on postoperative days 8 and 9 (p=0.0398), despite respiratory support by Nasal High Flow ^TM^ and an increase in FiO_2_. Paired t-tests were performed with EZR (Saitama Medical Center, Jichi Medical University, Saitama, Japan, v 1.61). EZR is a graphical user interface for R (The R Foundation for Statistical Computing, Vienna, Austria). More precisely, it is a modified version of R commander designed to add statistical functions frequently used in biostatistics. ※1 SpO_2_ was recorded every 30 min. ※2 The FiO_2_ of the nasal cannula was determined to be 60% based on a study by Kuluz et al. [12].

He was kept on sedation with intubation as per the original plan with postoperative management performed mainly by pediatric cardiologists. Furosemide (0.1 mg/kg/h) was used to maintain urine volume. His wound was examined daily by the attending otolaryngologist. The otolaryngologists observed the wound using a pharyngolaryngeal fiber to check for bleeding. They also observed the crusts that formed after the hemostatic procedure, peeled off the crusts, closely monitored the progress of each stage of epithelialization, and shared these observations with the treatment team. We predicted that edema would easily occur in and around the surgical wound, as his head and neck area was easily edematized by a slight volume overload. Furosemide (0.1 mg/kg/h) was used to prevent edema around the wound. Despite the furosemide edema prevention, edema developed in the pharyngeal laryngeal wall on the third postoperative day to the extent that the otolaryngologist was unable to observe the surgical wound. This pharyngeal edema far exceeded our expectations and the planned extubation was postponed to postoperative day 7 (Figure 2B). After extubation, 4 L/min of oxygen was administered through the nasal cannula for 2 days, but he suffered from difficulty breathing and was supported by HFNC. Postoperative hemorrhage was thought to be a risk not only within six hours after surgery but also from postoperative days 7 to 10, so we cautiously waited for the tonsillectomy wound to completely heal before restarting aspirin and warfarin, which occurred on postoperative day 15. His clinical course in the ICU highlighted a significant required oxygen concentration increase compared to before the surgery (Figure 3). Focusing on the SpO_2_ and FiO_2_ values preoperatively and after seven days postoperatively when the patient was extubated, a decrease in SpO_2_ was observed in the postoperative period. Furthermore, the FiO_2_ required to maintain a comparable SpO_2_ increased from postoperative day 10. On postoperative day 7, we suspected atelectasis or postoperative pulmonary edema due to prolonged sedation and intubation management, but there were no obvious abnormal findings on the chest X-ray. Eleven days after the Glenn procedure his SpO_2_ dropped to 60% and an emergency BTS was done. We thought that if the BTS was occluded, SpO_2_ would deteriorate to 60% under pure oxygen postoperatively, which it did not. Hence, we considered the possibility of a stenosis of the BTS but did not actively suspect complete occlusion. However, because of the prolonged increase in oxygen demand, we gradually began to suspect BTS obstruction or partial obstruction, and on the twenty-third postoperative day, we confirmed BTS obstruction by angiography. The endovascular approach to release the occlusion was unsuccessful. He underwent BTS reconstruction at postoperative day 35 and was discharged 88 days after the tonsillectomy.

## Discussion

As mentioned earlier, the frequency of post-tonsillectomy hemorrhage is approximately 0.5 to 18% [9], with life-threatening postoperative hemorrhage occurring in up to 5% of pediatric patients [10]. On multivariate analysis, Miller et al. concluded that CHD was not associated with an increased risk of occurrence of an unscheduled hospitalization or emergency room visit within 30 days of discharge after tonsillectomy. Their study included 244,597 patients, of which 858 had CHD [13] with the caveat that the previous surgical history of the CHD patients was not detailed. However, we do not know how many of the cases they studied had Glenn circulation nor how many of the cases they investigated had BTS added after the Glenn procedure.

In our case, the pulmonary artery flow from the Glenn anastomosis was incapable of maintaining his circulation so a BTS procedure was done. The mean pressure of his superior vena cava was as high as 17 mmHg before the tonsillectomy, and the pressure of his head and neck veins were similarly simultaneously high. The higher the pressure in a vein, the greater the amount of bleeding when that vein breaks down. Stated another way, bleeding can occur even with minor damage to a vein that would not bleed under normal venous pressure. Such high venous pressure put him at a greater risk of bleeding from the wound after the tonsillectomy. His usual SpO_2 _was about 80% so any further decrease could be fatal. We were aware of evidence that even patients on aspirin have a high incidence of BTS embolic occlusion (22.7%) [14] and that this risk could increase if aspirin and warfarin were stopped. We felt that reducing the risk of hemorrhage was more important than the potential for thrombus formation so we decided to discontinue aspirin and warfarin treatment five days before the operation.

Here, we discuss each of the factors that contributed to the BTS occlusion in this case. First, in the present case, no clear site of stenosis was identified for the shunt flow path. In addition, the graft size of the BTS was 4 mm (>3.5 mm), which has a low risk of shunt occlusion according to Soueidi et al. [15]. Unfortunately, however, his BTS was occluded. This might be attributed to another mechanical factor. In a patient with BTS alone responsible for pulmonary blood flow, BTS blood flows to the left and right pulmonary arteries. In our case, the BTS and superior vena cava were responsible for pulmonary blood flow. His preoperative angiography showed that blood from the superior vena cava flowed to the right pulmonary artery and blood from the BTS predominantly flowed to the left pulmonary artery increasing flow resistance in the BTS. This anatomical factor could be one of the reasons for the occlusion of the 4 mm graft-size BTS.

Sahoo et al. reported that low hematocrit (<45%) is associated with a lower risk of BTS occlusion [16]. In our case, hematocrit vacillated after the tonsillectomy; 38.2% at the volume overload and 50.3% at the volume underload. Considering the risk of thrombosis, we should have adjusted his fluid balance so that his hematocrit was less than 45%. However, his Glenn circulation was problematic, with a preoperative high mean superior vena cava pressure of 17 mmHg. His fluid balance was difficult to control, and his face would swell when the fluid volume in his body increased slightly. Postoperatively, the wound was swollen with edema to the point that it could not be examined by otolaryngologists. To control the head and neck edema, it was necessary to drain the fluid with furosemide. It was therefore difficult to keep the hematocrit low. His high hematocrit would have put him at high risk for BTS obstruction. In retrospect, phlebotomy may have been a way to lower hematocrit without worsening edema.

Finally, as patient-specific variables, Guzzetta et al. emphasize that low shunt blood flow resistance is important for shunt patency [17]. Our patient had pulmonary hypertension; pulmonary vascular resistance in the peripheral pulmonary arteries would have been high. Endothelial injury (one of Virchow's triads) is associated with shunt obstruction [17]. He was also exposed to increased pulmonary blood flow until five months of age, which may have caused endothelial injury such as necrosis and vasculitis in his peripheral pulmonary arteries [18]. His pulmonary blood flow was controlled by pulmonary artery banding (PAB), but he still suffered from pulmonary hypertension thereafter. This fact suggested that his peripheral pulmonary artery lesions might have been prolonged. Thus, such endothelial injury could also have been a risk for BTS occlusion.

Considering all the variables at play, his risk of BTS occlusion was high. Part of our team insisted that heparin should be administered the day after surgery. Since heparin can be quickly antagonized by protamine [19], they argued that protamine should be administered as soon as postoperative hemorrhage occurs. Some reports have shown that a 4 mm BTS rarely occludes [15,20], hence we felt the risk was not high and chose to focus on the prevention of a postoperative hemorrhage. In retrospect, it may have been better to restart anticoagulation therapy immediately after the surgery.

A paired t-test showed that SpO_2_ was statistically lower on postoperative days 10 to 23 (after respiratory support with a high-flow nasal cannula) than on postoperative days 8 to 9 (after extubation and oxygenation with a nasal cannula). We do not know when the BTS occlusion occurred. However, his oxygenation capacity gradually worsened over time postoperatively, according to statistical analysis. This worsening can also be seen in the FiO_2_ adjustments shown in Figure 3. Thus, if heparin had been administered as soon as possible after the end of surgery, the deterioration of his oxygenation capacity could have been prevented.

Although there is no clear evidence as to when anticoagulation should be resumed, we still believe that anticoagulation should be stopped on the day of surgery to stabilize the crust. However, anticoagulation should be resumed the day after surgery and during intubation. Intubation provides a completely safe airway, so if postoperative hemorrhage had occurred, we could have kept airway patency and avoided asphyxiation. Therefore, anticoagulation with heparin should have been administered at least during intubation.

## Conclusions

We report our experience of anesthetic management of pediatric tonsillectomy of a child with post-Glenn and BTS dependency. In our case, pediatric cardiologists, cardiac surgeons, otolaryngologists, and anesthesiologists participated in the conference. Postoperative hemorrhage was avoided successfully, which we believe was a result of daily monitoring of the wound by otolaryngologists and the replacement of aspirin and warfarin with heparin five days before the surgery. His surgical wound took 15 days to stabilize the crust, peel off the crust, and completely epithelialize. Thus, we resumed anticoagulation therapy 15 days after surgery, and unfortunately, his BTS developed thrombotic occlusion and required a planned BTS reconstruction operation, which was done 35 days after the initial surgery. As mentioned previously, BTS occlusion rarely occurs with graft size greater than 3.5 mm. However, as in our case, a combination of mechanical, chemical, and patient-specific factors can cause occlusion even in grafts larger than 3.5 mm.

Anesthesiologists must weigh the risks of severe hemorrhage against potential occlusion when considering whether or not to stop anticoagulation treatment. The timing of their resumption must also be carefully considered. During intubation, there is a relatively low risk of fatal asphyxiation due to postoperative bleeding. It is hard to draw conclusions from a single case, but it is our opinion that anticoagulation with heparin should be administered during intubation beginning the day after surgery to reduce the risk of BTS occlusion.

## Figures and Tables

**Figure 4:**
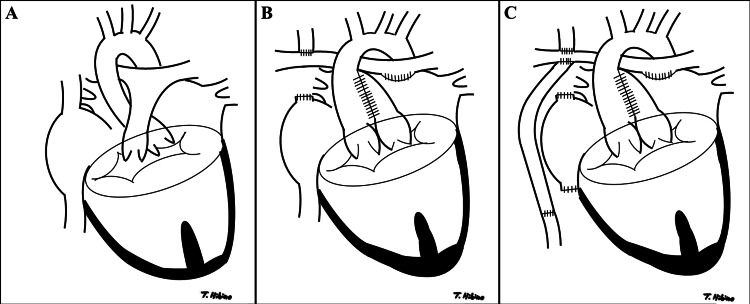
Heart structure and treatment plan for this case A: Figure A shows the patients' heart before treatment. The diagnosis was an atrioventricular septal defect. Blood from the right atrium and the left atrium were mixed and entered the ventricles through the common atrioventricular valve. The left ventricle was smaller than the right ventricle, and the unusual shape of the common atrioventricular valve was a relative contraindication for biventricular repair. B: Figure B shows the Damus-Kay-Stansel anastomosis and the Glenn anastomosis. A single ventricle reconstruction was planned for him. The aorta and pulmonary artery exiting the ventricle would be united using the Damus-Kay-Stansel procedure. The pulmonary artery flow was supplied by the superior vena cava using the Glenn procedure. C: Figure C shows the ideal final form of his treatment plan. The Fontan anastomosis has been added to Figure B. A series of single ventricle reconstructions is completed with the Fontan procedure, in which the inferior vena cava and pulmonary artery are connected by an epicardial conduit. This allows all systemic venous blood to flow to the lungs and all arterial blood to flow systemically. Unfortunately, after the Glenn and Dams-Kay-Stansel procedures, he had insufficient pulmonary blood flow. This was due to high pulmonary vascular resistance, and the Fontan procedure was not possible. Image credits: Tokimitsu Hibino
